# Age Dependency of GLI Reference Values Compared with Paediatric Lung Function Data in Two German Studies (GINIplus and LUNOKID)

**DOI:** 10.1371/journal.pone.0159678

**Published:** 2016-07-20

**Authors:** Anke Hüls, Ursula Krämer, Monika Gappa, Christine Müller-Brandes, Tamara Schikowski, Andrea von Berg, Barbara Hoffmann, Antje Schuster, Matthias Wisbauer, Claudia Flexeder, Joachim Heinrich, Holger Schulz, Dietrich Berdel

**Affiliations:** 1 IUF-Leibniz Research Institute for Environmental Medicine, Düsseldorf, Germany; 2 Marien Hospital Wesel, Children’s Hospital and Research Institute, Wesel, Germany; 3 Medical School of Hanover, Department of Anaesthesiology and Intensive Care Medicine, Hanover, Germany; 4 Medical Faculty, Heinrich-Heine University of Düsseldorf, Düsseldorf, Germany; 5 Heinrich-Heine University, University Children’s Hospital, Düsseldorf, Germany; 6 Institute of Epidemiology I, Helmholtz Zentrum München - erman Research Center for Environmental Health, Munich, Germany; 7 Comprehensive Pneumology Center Munich (CPC-M), Member of the German Center for Lung Research, Munich, Germany; University of Giessen Lung Center, GERMANY

## Abstract

A hallmark of the newly published GLI (Global Lungs Initiative) spirometric reference values is their "all-age" (3-95yr) predictive power, accomplished by incorporating non-linear age dependencies into modelling parameters. This modelling strategy is especially promising for the age range of puberty; however, the performance of GLI-values for adolescents is currently unknown. We calculated GLI-based z-scores for children/adolescents without apparent respiratory diseases from two different German studies, LUNOKID (N = 1943, 4–19 years) and GINIplus (N = 1042, 15 years) and determined the goodness of fit for specific age groups. We defined fit sufficient if the absolute mean of z-scores was <0.5. For children (<10yr) the mean GLI-based z-scores for FEV_1_ and FVC reached a good fit with mean z-scores for FEV_1_ between -0.11 and 0.01 and mean z-scores for FVC between 0.01 and 0.16, but larger deviations were observed in adolescents, especially boys (mean z-score -0.58 for FEV_1_ and -0.57 for FVC in GINIplus). The fit for FEV_1_/FVC was sufficient. GLI reference values provided reasonable estimates for the individuals enrolled in our studies, which span the age range of lung growth and development. However, we found that GLI-predictions overestimated lung volumes, especially those for German adolescent boys, which may, left unrecognised, lead to erroneous diagnosis of lung disease. Caution should be taken when applying these reference values to epidemiologic studies.

## Introduction

Spirometry is the most frequently used method with which to routinely assess lung function in clinical and research settings. However, the interpretation of results is crucially dependent on the use of appropriate reference data [1,2]. The use of historic reference values e.g. those developed by Zapletal *et al*. [3] in the 80s, although still in clinical use, are no longer the most appropriate [4,5].

Recently, the Global Lung Initiative (GLI) published multi-ethnic reference values with which to interpret spirometric results. These provide mean values and values for the lower limit of normal (LLN) from preschool to old age [5], achieved by including non-linear age dependencies, which are especially important for adolescents where the relationship between lung volume and height depends on maturity [6].

Global comparisons of GLI reference values with observations made from national studies have been published [7–10]. Using absolute mean z-scores of less than 0.5 to indicate sufficient fit, Hall and Thompson [7–10] showed that the newly published reference values were applicable to Australasian Caucasians. Likewise, overall mean GLI-based z-scores for the datasets (N>150) included in its reference population did not exceed 0.5 [11], indicating sufficient fit.

Global comparisons may however be misleading; a small global absolute mean of GLI based z-scores across ages does not necessarily preclude higher deviations for certain age groups. Systematic deviations from the proposed age relationship in GLI reference equations have already been investigated for adults, with two Scandinavian [12,13], and one Japanese report [14]. Thus far, only one study has investigated systematic z-score trends over age, for children and adolescents. Unfortunately that study predated the introduction of the GLI indices, and used historic reference equations instead [6]. Consequently, equivalent investigations for children and adolescents using GLI reference equations are still lacking.

Therefore, we aimed to investigate the fit between GLI derived means and LLN values during specific age periods in childhood and adolescence, by taking advantage of two independent German studies. The first incorporated children and adolescents from the LUNOKID-study (LUng function NOrmal values for KIDs in Germany), part of the original GLI reference population [11]. The second involved 15-year old adolescents from the GINIplus study (German Infant Nutritional Intervention plus environmental and genetic influences on allergy development study), which was not included in the GLI reference population.

## Materials and Methods

### Study design and study population

The first study analysed is the LUNOKID-study which aimed to generate new German-specific reference values for spirometry in children and adolescents. Details on the design, recruitment, lung function measurements, and quality criteria have been published [4,15–17]. Briefly, Caucasian children and adolescents from randomized kindergartens and schools, aged 4 to 19, were recruited from three cities in Germany (Wesel, Düsseldorf, Hannover) in 2008/2009. The children were tested in their schools and kindergartens, with parents completed a questionnaire on the respiratory health of their child and influencing factors (e.g. smoking, preterm birth).

The second study is the 15-year follow-up of GINIplus, which was designed to prospectively investigate influences on allergy development in a population based birth-cohort study, with nutritional intervention included for predisposed infants in the first months of life. Details on the design, recruitment and follow-up of this study have been published [18,19]. Briefly, a total of 5991 new-borns were recruited in obstetric clinics in Munich and Wesel, Germany, between September 1995 and July 1998. Follow-up occurred at the age of one, two, three, four, six, ten, and 15 years of age. Our analyses are based on lung function data from the 15-year follow-up.

Both studies were approved by the relevant ethics committees (Ethikkommission der Ärztekammer Nordrhein and Ethikkommision der Bayerischen Landesärztekammer) with written informed consent obtained from the parents of all participants.

### Lung function measurement

Detailed descriptions of the lung function measurements performed in LUNOKID and GINIplus have been published elsewhere [15,20]. Briefly, in both studies spirometry was carried out using an EasyOne handheld device (ndd-Medizintechnik AG, Zürich, Switzerland). Only non-smoking children with acceptable lung function according to the ATS/ERS criteria including visual control were considered for the analysis (N = 3205 in LUNOKID and N = 1628 in GINIplus) [1,2,15,20]. Furthermore we restricted our analyses to children/adolescents without apparent respiratory disease. Children with physician diagnosed asthma, spastic bronchitis or an infection on the day of investigation were excluded. Additionally, we excluded children/adolescents with a lower respiratory tract infection in the preceding 6 weeks from the LUNOKID reference population [17] and adolescents with a respiratory tract infection in the preceding week from the GINIplus population. In a sensitivity analysis, adolescents with a respiratory tract infection in the preceding 2–4 weeks were additionally excluded from the GINIplus dataset. Further we performed a sensitivity analysis in the GINIplus cohort, in which we excluded participants with a nutritional intervention in the first months of life. In additional sensitivity analyses we excluded from both cohorts participants who were exposed to passive smoke at home, who had atopic eczema or hayfever and who were born preterm (<37 weeks).

### Statistical analyses

The GLI [5] and LUNOKID [4] reference values are both based on regression model LMS. For a detailed description of the LMS model see Cole and Green [21], or Rigby *et al*. [22]. Due to the definition of the LMS model, we can calculate GLI or LUNOKID based reference values for a participant’s expected mean (M), and additionally for a participant’s expected coefficient of variation (S), and skewness (L) [5]. Given M, S and L, the standardized z-scores can be calculated as

$$z\;=\;\frac{{\left(\frac{Y}{M}\right)}^{L}-1}{L\times \;S}$$

where Y is the observed FEV_1_, FVC, and FEV_1_/FVC. These z-scores have a standard normal distribution, and are independent of age for the reference populations. The coefficients needed for these calculation are provided in Quanjer *et al*. [5] (GLI), or Hüls *et al*. (LUNOKID) [4].

Prior to analyses, we checked datasets for outliers as our statistical tests are sensitive to these [11]. For the LUNOKID reference data we separately checked the FEV_1_, FVC, and FEV_1_/FVC values (adjusted for median height) for outliers, across 4 different age ranges; 3-<5 years, 5-<10 years, 10-<15 years, and 15-<20 years. Values more than 1.5 IQR (interquartile range) beneath, or above the lower and upper quartiles respectively, were excluded. The same procedure was also applied to the GINIplus data.

To investigate age-specific fits for the GLI reference values to measurements completed for LUNOKID, we divided our data into 4 different age groups, two were for children aged 3-<5 years, and 5-<10 years, and two were for adolescent groups of 10-<15 years, and 15-<20 years. Should the reference values prove to be a perfect fit for the study population, then the mean would be zero, and the standard deviation one, for every age group. Following the example of Hall [7] and Thompson [10], an absolute mean z-score of less than 0.5 was chosen to indicate sufficient fit. Two one-sided tests (TOST) for equivalence were conducted, and we tested our null hypothesis of a mean z-score outside of the interval [-0.5, 0.5]. If rejected, then the fit is sufficient. Furthermore, we calculated the percentage of observed lung function values lying below the predicted lower limit of normal (LLN). With a perfect fit, 5% of values should lie below the LLN. Additionally, we plotted the GLI based z-scores of the LUNOKID observations with age, together with the smoothed mean and 5^th^ percentile using GAMLSS in R [23]. With a perfect fit to age, we would expect the smoothed mean to be constant zero, and the smoothed 5^th^ percentile to be constant -1.64 (LLN). All analyses where conducted using R 3.1.1 [24].

## Results

### Description of the study population

The LUNOKID study population comprised 3205 participants with a mean age of 11.2 years (48.6% males), with the GINIplus study population comprising 1628 participants with a mean age of 15.3 years (47.2% males) (Table 1). With the exception of wheezy bronchitis, the prevalence of asthma and respiratory tract infections were comparable for both studies. In total, after excluding participants with apparent respiratory disease, our study population comprised 1943 (1922 without outliers) lung healthy participants from LUNOKID, and 1042 (1032 without outliers) from GINIplus, this is slightly more than 60% of the original study population. A more detailed description of these lung healthy participants is shown in Table 2. Table 2 further gives an overview about additional exclusion criteria that were used in the sensitivity analyses.

**Table 1:** Description of study populations (LUNOKID and GINIplus).

	LUNOKID	GINIplus
Total N*	3205	1628
Age range, mean (min-max)	11.2 (4.0–19.0)	15.3 (14.7–16.8)
Sex, n (%) male	1557 (48.6%)	769 (47.2%)
BMI, mean (sd)	19.4 (4.9)	20.9 (3.2)
Asthma, n (%)	279 (8.7%)	190 (11.7%)
Wheezy bronchitis, n (%)	671 (20.9%)	76 (4.7%)
Airway infection (day), n (%)	440 (13.7%)	183 (11.2%)
Airway infection (last weeks^¶^), n (%)	241 (7.5%)	208 (12.8%)
Lung Healthy, n (%)	1943 (60.6%)^†^	1042 (64.0%)^#^

*: non-smokers with acceptable lung function according to the ATS/ERS criteria including visual control;

^¶^: LUNOKID: last 6 weeks; GINIplus: last week;

^†^: without asthma, wheezy bronchitis, infection (day and/or last 6 weeks);

^#^: without asthma, wheezy bronchitis, infection (day and/or last week)

**Table 2:** Description of the lung healthy study populations (LUNOKID and GINIplus). Further exclusion criteria for sensitivity analyses.

	LUNOKID	GINIplus
Total N^¶^	1943	1042
Age range, mean (min-max)	11.0 (4.0–19.0)	15.3 (14.7–16.8)
Sex, n (%) male	901 (46.4%)	466 (44.7%)
Exposure to passive smoke at home, n (%)	656 (33.8%)	125 (12.0%)
Ever atopic eczema, n (%)	202 (10.4%)	189 (18.1%)
Ever hayfever, n (%)	119 (6.1%)	167 (16.0%)
Neither atopic eczema nor hayfever, n (%)	1622 (83.5%)	721 (69.2%)
Born preterm (<37 weeks), n (%)	181 (9.3%)	23 (2.2%)
Infection (last 2–4 weeks), n (%)	*n*.*a*.	121 (11.6%)
Intervention group*, n (%)	*no intervention*	490 (47.0%)

*: Intervention group: Nutritional intervention for predisposed infants in the first months of life due to study design;

^¶^: Lung healthy participants that were used for the main analysis (compare Table 1)

### LUNOKID data compared to the GLI reference values

**Fig 1:**
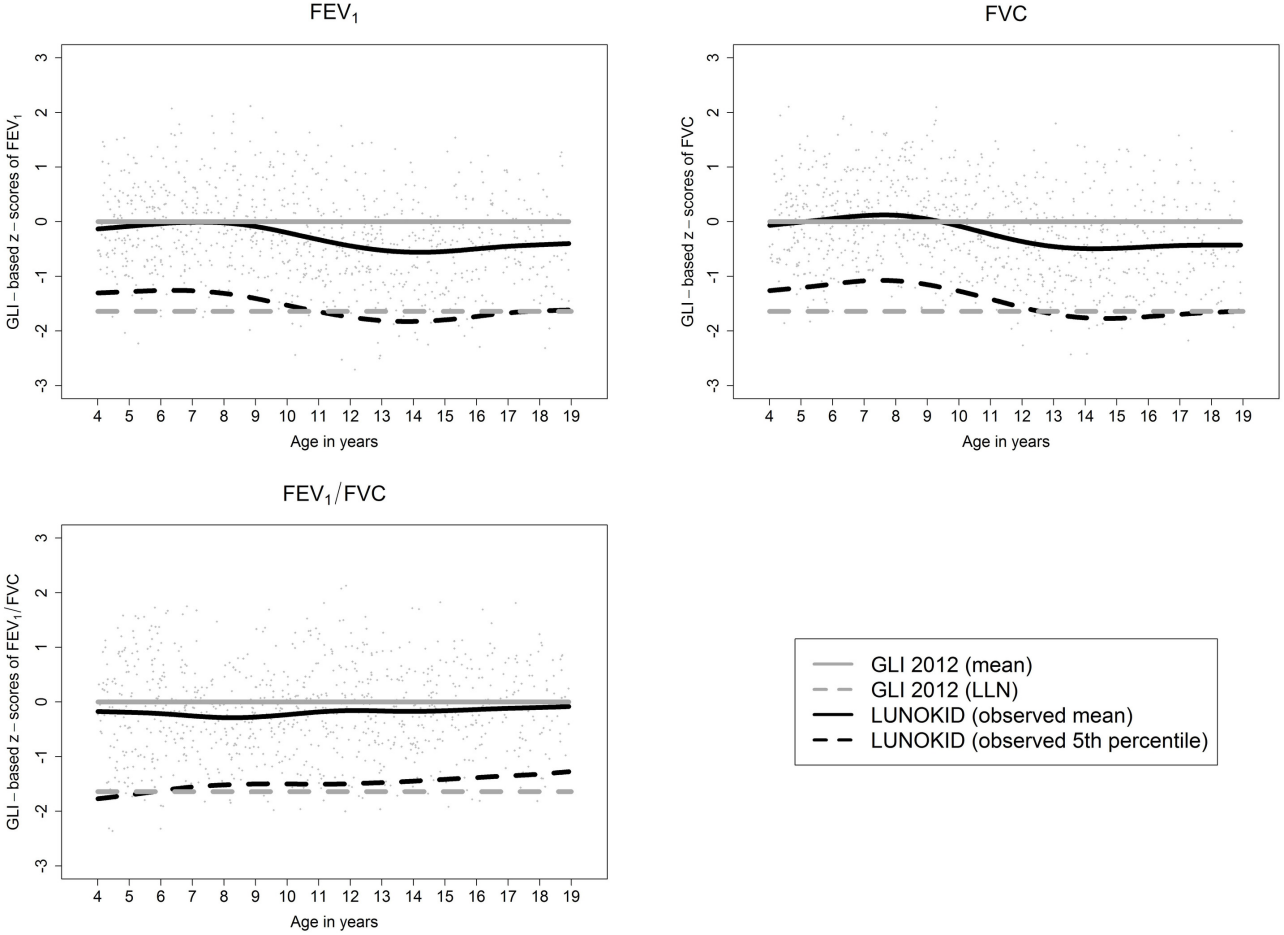
Comparison of the GLI-z-scores (mean and lower limit of normal (LLN)) with these observed in boys from the LUNOKID data.

Table 2 shows means and standard deviations of the GLI-based z-scores for FEV_1_, FVC and FEV_1_/FVC for our four age categories in LUNOKID. A graphical representation of the GLI based z-scores over age is given in Fig 1 for boys, and in S1 Fig for girls. The mean GLI-based z-scores for FEV_1_ and FVC reached a good fit for children below 10 years of age and decreased with age. From the age of 10 onwards z-scores for FEV1 reached values of -0.2 to -0.3 and z-scores for FVC reached values of -0.3 to -0.4. The effect was larger for boys than for girls. For the 315 boys in the ≥10 to <15 age group, the mean of the GLI based z-scores for FEV1 was -0.48, with a standard deviation of 0.81 (Table 3). Consequently, the null hypotheses of a deviation ≥ 0.5 could not be rejected (indicating insufficient fit). In comparison, the mean of the GLI based z-scores for the 334 girls in this age group was -0.20, with a standard deviation of 0.85 (indicating a sufficient fit). The fit of the mean GLI based z-scores for FEV1/FVC was sufficient for all age and gender groups.

**Table 3:** LUNOKID: GLI-based z-scores of all and of all male and female healthy children/adolescents grouped by age.

	Age group	N	mean (sd) ^#^	%≤LLN
**All**				
**FEV**_**1**_	≥4 & <6	290	-0.11 (0.76) ***	2.1%
	≥6 & <10	561	0.01 (0.82) ***	2.0%
	≥10 & <15	649	-0.33 (0.84) ***	4.9%
	≥15 & <19	421	-0.40 (0.77) **	6.2%
	All	1921	-0.21 (0.82) ***	3.9%
**FVC**	≥4 & <6	286	0.01 (0.76) ***	1.4%
	≥6 & <10	560	0.16 (0.76) ***	0.4%
	≥10 & <15	651	-0.23 (0.81) ***	3.5%
	≥15 & <19	425	-0.30 (0.81) ***	4.7%
	All	1922	-0.09 (0.81) ***	2.5%
**FEV**_**1**_**/FVC**	≥4 & <6	291	-0.24 (0.98) ***	7.6%
	≥6 & <10	553	-0.31 (0.83) ***	5.2%
	≥10 & <15	641	-0.16 (0.83) ***	2.3%
	≥15 & <19	424	-0.15 (0.85) ***	2.8%
	All	1909	-0.21 (0.86) ***	4.1%
**Males**				
**FEV**_**1**_	≥4 & <6	147	-0.11 (0.74) ***	2.0%
	≥6 & <10	268	0.02 (0.81) ***	1.5%
	≥10 & <15	315	-0.48 (0.81)	6.0%
	≥15 & <19	160	-0.41 (0.79)	5.0%
	All	890	-0.26 (0.82) ***	3.8%
**FVC**	≥4 & <6	146	-0.02 (0.78) ***	1.4%
	≥6 & <10	267	0.15 (0.77) ***	0.0%
	≥10 & <15	316	-0.37 (0.79) **	4.1%
	≥15 & <19	160	-0.40 (0.83)	5.6%
	All	889	-0.16 (0.83) ***	2.7%
**FEV**_**1**_**/FVC**	≥4 & <6	147	-0.15 (0.98) ***	4.8%
	≥6 & <10	261	-0.28 (0.75) ***	3.8%
	≥10 & <15	311	-0.15 (0.83) ***	2.6%
	≥15 & <19	160	-0.10 (0.77) ***	2.5%
	All	879	-0.18 (0.83) ***	3.3%
**Females**				
**FEV**_**1**_	≥4 & <6	143	-0.11 (0.79) ***	2.1%
	≥6 & <10	293	0.01 (0.82) ***	2.4%
	≥10 & <15	334	-0.20 (0.85) ***	3.9%
	≥15 & <19	261	-0.38 (0.76) *	6.9%
	All	1031	-0.17 (0.82) ***	4.0%
**FVC**	≥4 & <6	140	0.05 (0.73) ***	1.4%
	≥6 & <10	293	0.17 (0.75) ***	0.7% **
	≥10 & <15	335	-0.09 (0.81) ***	3.0%
	≥15 & <19	265	-0.24 (0.79) ***	4.2%
	All	1033	-0.04 (0.79) ***	2.4% **
**FEV**_**1**_**/FVC**	≥4 & <6	144	-0.34 (0.97) *	10.4%
	≥6 & <10	292	-0.34 (0.90) **	6.5%
	≥10 & <15	330	-0.17 (0.82) ***	2.1%
	≥15 & <19	264	-0.18 (0.89) ***	3.0%
	All	1030	-0.25 (0.89) ***	4.8%

^#^: Two one-sided tests (TOST) for equivalence. Fit is sufficient, if the null-hypothesis of a mean z-score outside of the interval [-0.5, 0.5] is rejected

(*:p<0.05, **: p<0.01, ***: p<0.001).

For all of the age groups and lung function indices, the standard deviations of the GLI based z-scores were less than one, which influenced the fit of the GLI based LLN. Only 2.1% (1.4%) of the values for 4- to <6-year old children, and 2% (0.4%) of the values for 6- to <10-year olds, were below the LLN for FEV_1_ (FVC). This effect was not seen in the adolescent groups because the influence of their smaller standard deviation was offset by lower mean values.

### GINIplus data compared to the GLI and LUNOKID reference values

Table 4 shows the means and standard deviations of the GLI-based z-scores for FEV_1_, FVC, and FEV_1_/FVC, measured in GINIplus. For FEV_1_ and FVC, these means were below -0.5 indicating insufficient fit, with deviation more pronounced for boys than girls. Although the standard deviation was smaller than the expected 1, more adolescents than the expected 5% fell beneath the LLN. Again, this effect was more pronounced in males (15.0%≤LLN for FEV_1_ and 14.3%≤LLN for FVC) than females (both ≈8%). The difference in mean GLI-based z-scores between males and females was significant for FVC (t-test, p = 0.008). In contrast the mean GLI based z-scores for FEV_1_/FVC values were approximately zero, with standard deviations approximating one, indicating a sufficient fit.

**Table 4:** GINIplus: GLI- and LUNOKID-based z-scores of all and of all male and female healthy participants.

		GLI		LUNOKID	
	N	mean (sd) ^#^	%≤LLN	mean (sd) ^#^	%≤LLN
**All**					
FEV_1_	1032	-0.58 (0.87)	11.5% ***	0.02 (1.12) ***	7.3% *
FVC	1029	-0.57 (0.85)	10.9% ***	-0.12 (1.06) ***	8.0% **
FEV_1_/FVC	1038	-0.04 (0.97) ***	5.4%	0.28 (1.17) ***	6.2%
**Males**					
FEV_1_	461	-0.62 (0.91)	15.0%	0.07 (1.19) ***	9.1%
FVC	461	-0.65 (0.88)	14.3%	-0.13 (1.08) ***	9.1%
FEV_1_/FVC	464	-0.03 (0.94) ***	4.1%	0.34 (1.20) **	5.6%
**Females**					
FEV_1_	571	-0.55 (0.84)	8.8% *	-0.02 (1.05) ***	5.8%
FVC	568	-0.51 (0.82)	8.1% *	-0.10 (1.04) ***	7.0%
FEV_1_/FVC	574	-0.06 (0.98) ***	6.4%	0.23 (1.14) ***	6.6%

^#^ Two one-sided tests (TOST) for equivalence. Fit is sufficient if the null-hypothesis of a mean z-score outside of the interval [-0.5, 0.5] is rejected

(*: p<0.05, **: p<0.01, ***: p<0.001).

Table 4 also shows the data following application of the LUNOKID derived reference values to GINIplus. Means and standard deviations of the LUNOKID based z-scores for observations from GINIplus indicated a sufficient fit for males and females.

### Sensitivity analyses: Use of stricter exclusion criteria

In the sensitivity analyses we excluded beside participants with asthma, wheezy bronchitis or infection (day and/or last 6 weeks) also participants who were exposed to passive smoke at home, who ever had atopic eczema or hayfever and who were born preterm (<37 weeks) (Table 5). These sensitivity analyses showed that our previous findings were not influenced by the exclusion criteria we chose in our main analysis. In a further sensitivity analysis in the GINIplus cohort, we additionally excluded participants with a respiratory tract infection in the last 2–4 weeks and participants with a nutritional intervention in the first months of life (S1 Table). These exclusion criteria had again no impact on our findings thus confirming our previous conclusions.

**Table 5:** GLI-z-scores in GINIplus and LUNOKID in all lung healthy children (All) and in subgroups after using stricter exclusion criteria.

	LUNOKID			GINI		
	N	mean (sd) ^#^	%≤LLN	N	mean (sd) ^#^	%≤LLN
**All**						
FEV_1_	1921	-0.21 (0.82) ***	3.9%	1032	-0.58 (0.87)	11.5% ***
FVC	1922	-0.09 (0.81) ***	2.5% ***	1029	-0.57 (0.85)	10.9% ***
FEV_1_/FVC	1909	-0.21 (0.86) ***	4.1%	1038	-0.04 (0.97) ***	5.4%
**No passive smoke**						
FEV_1_	1267	-0.20 (0.83) ***	3.9%	890	-0.56 (0.87)	11.1% ***
FVC	1273	-0.10 (0.82) ***	2.4% ***	888	-0.56 (0.85)	10.5% ***
FEV_1_/FVC	1266	-0.18 (0.86) ***	3.6%	896	-0.01 (0.96) ***	4.6%
**No atopic eczema / hayfever**						
FEV_1_	1600	-0.21 (0.83) ***	4.0%	713	-0.59 (0.87)	10.8% ***
FVC	1602	-0.09 (0.81) ***	2.5% ***	714	-0.57 (0.85)	10.2% ***
FEV_1_/FVC	1596	-0.22 (0.85) ***	4.1%	717	-0.05 (0.96) ***	5.0%
**Not born preterm (≥37 weeks)**						
FEV_1_	1725	-0.21 (0.82) ***	3.6%	993	-0.58 (0.87)	11.5% ***
FVC	1727	-0.10 (0.80) ***	2.7% ***	991	-0.57 (0.85)	11.0% ***
FEV_1_/FVC	1715	-0.20 (0.86) ***	3.9%	999	-0.05 (0.96) ***	5.4%

^#^ Two one-sided tests (TOST) for equivalence. Fit is sufficient if the null-hypothesis of a mean z-score outside of the interval [-0.5, 0.5] is rejected

(*: p<0.05, **: p<0.01, ***: p<0.001).

## Discussion

We compared the age dependency of the GLI reference values with observations from the German LUNOKID study, and demonstrated a sufficient fit for children up to the age of ten years. However, for older children and adolescents, we found an ever-increasing divergence in volume measurements, with an insufficient fit for boys of 10 to 15 years of age. The same was true for adolescents in the GINIplus study, with a mean age of 15 years. However, a sufficient fit was found for FEV_1_/FVC. Furthermore, the standard deviations for the z-scores for FEV_1_ and FVC observed in the German studies were generally less than one.

Until now, several studies have compared GLI reference values with national reference data. The study conducted by Hall *et al*. [7] concluded that the GLI indices were applicable for Caucasians from Australia and New Zealand (n = 2066, age: 4–80 years). In our previous study on the applicability of the GLI reference values for longitudinal data of elderly women from the German SALIA study, we came to the same conclusion (n = 1726, mean age of 54 years at baseline examination) [25]. In contrast, Ben Saad *et al*. found that the GLI reference values were of less value for adult North African Caucasians in Tunisia (n = 489, age: 18–60 years) [8]. Age specific comparison studies have also been completed. For example, Backman *et al*. [12] evaluated the GLI values for a Swedish reference population (N = 501, age: 22–91) and found that mean GLI based z-scores for lung volume systematically increased with age, with the effect magnified for men versus women. The same trend was found for a population of Finish adults (n = 1000, age: 18–83) [13]. Again the z-scores for volume increased with age, reaching values of approximately 0.8 in men older than 70 years of age. Increasing z-scores between the ages of 40 and 80 were also found in a Japanese reference group (n = 20,336, age = 17–95) [14]. However, in contrast to the Scandinavian studies, the best fit was with the oldest age group, with z-scores of less than -0.5 occurring in the youngest group. For the Japanese study, secular changes in relative leg length (which was higher for the younger age group), partially explained the trend of deviation from the height-based GLI reference values; a similar effect could eventually explain the trends found in the Scandinavian studies, where height and age were also negatively correlated.

Similar age-specific comparison studies for GLI reference data during adolescence are however missing. A single study, which predated GLI and used older reference data, revealed a U-shaped deviation with the lowest z-scores obtained for males during puberty [6].

We found the lowest GLI based z-scores for FEV_1_ and FVC after the age of 10, particularly for males, which roughly coincides with the beginning of their growth spurt during puberty [26]. In contrast, the fit for FEV_1_/FVC was found to be sufficient. These data suggest a modifying effect for adolescents derived from a basic physiologic parameter. It is well appreciated that adolescents have a lower lung volume than expected for their height [27–29] during the time when growth is most rapid. However, height development differs even between Caucasian populations, especially for adolescents with respect to final height reached, as for the timing of the beginning of the growth spurt. Whereas median height up until the age of ten does not vary greatly between European populations, thereafter, values for Northern and Southern Europeans diverge. Median height is, for instance, 176 cm in an 18 year old Italian male compared to 182 cm for a Dutch male (and 180 cm for a German male) [30]. The age at puberty on the other hand is earlier in Italy than in the Netherlands [26]. GLI categorises Caucasians as a single ethnic group, and reflects mean age and height related lung development, but without accounting for age related deviations for specific Caucasian groups. This may lead to age and population specific mean deviations, and offers a tentative explanation for why the German LUNOKID based reference values fit the mean values of German adolescents from GINIplus better than the GLI reference values.

Whether the mean differences observed are clinically relevant is controversial. We adopted the criterion introduced by Hall [7] and Thompson [10], who considered a deviation of 0.5 z-scores (1/2 standard deviation) to be clinically relevant. We found that at least 50% of German adolescent reference males presented a lung volume below -0.5 z-scores. However, whether the absolute mean of the z-score is 0.4, 0.5, or 0.6, mostly will not influence diagnostic decision-making. Of more importance is the LLN, which is used as a diagnostic cut-off in clinical practice.

The height of the LLN is dependent on the mean and standard deviation. The standard deviations of our GLI based z-scores were smaller than expected. This may be partly explained by our strict visual quality control, which was applied in order to generate an accurate data set of normal values, suitable for clinical and epidemiological studies. Furthermore our study populations were ethnically homogeneous, with the same spirometric devices used for all measurements. These conditions contrast with those found for the combined reference data set for GLI, which is essentially a coalition of several different data sets. Thus the coefficient of variation from the GLI reference data set reflects inherent differences between centres due to their different measuring devices and population characteristics. The observed standard deviations of z-scores were also generally lower than expected in the Scandinavian reference populations [12,13]. Thus, even if the local and expected GLI means match, the local 5^th^ percentile may still lie above the GLI based LLN, which may have consequences for the diagnoses of lung diseases. Therefore, an impaired lung function of German children younger than 10 years of age, might not be recognized using the LLN of the GLI reference values. However, this is not a problem for children and adolescents older than 10 years of age. For these children the LLN of the GLI reference values fits well to the 5^th^ percentile observed in adolescents from the LUNOKID study.

Furthermore GLI based LLNs are not free from age or gender related bias when applied to German populations. German adolescents and males are more likely to be misdiagnosed with a lung disease when using GLI reference values than German children or females. This should be taken into account when assessing lung function for a German male adolescent. The quotient FEV1/FVC is free from this bias and should be the preferred metric in these cases. For clinical practice however it is important to keep in mind that a diagnosis should never be based on a single lung function test, other clinical parameters should be considered. Therefore, the differences in LLN should not lead to a false diagnosis if clinical decision-making is multifactorial.

The observed gender and age specific bias in GLI based z-scores are especially relevant for the application of reference values in epidemiological analyses. Such analyses sometimes use z-scores instead of the original spirometric measurements of lung volume to reduce the huge age and height related variance. LLNs are often used in epidemiology to objectively define obstructive or restrictive lung disease from lung function measurements. However if we applied GLI reference values to our GINIplus study, we would, for example, erroneously consider males to be more affected than females, and adolescents more so than children. For epidemiological applications it might therefore be better to use Nation/Germany-specific reference values (for Germany those derived from LUNOKID), where this bias is smaller, or to use no reference values at all but instead include age, height and gender in the analysis models.

## Conclusion

GLI reference values provide reasonable estimates for two independent German studies covering the age range of lung growth and development. Whilst universal availability and the continuity of reference values from childhood to adulthood, makes GLI reference data an attractive and valuable resource for clinical practice, we should bear in mind those patients for whom GLI- predictions may be slightly less accurate. In particular, the overestimates of lung volume in German adolescent boys could lead to erroneous diagnosis of lung diseases. We would advise an especially cautious approach when applying these reference values in the context of epidemiological studies.

## Supporting Information

S1 Fig Comparison of the GLI-z-scores (mean and lower limit of normal (LLN)) with these observed in girls from the LUNOKID data. (PDF)

S1 Table GINIplus (sensitivity analysis): GLI- and LUNOKID-based z-scores of all lung healthy 15-year old participants (All), participants without a respiratory tract infection in the last 2–4 weeks and participants with no nutritional intervention in the first months of life. (PDF)

## Abbreviations

LLN: lower limit of normal (5^th^ percentile)
CoV: coefficient of variation
sd: standard deviation
FEV_1_: forced expiratory volume in 1 s
FVC: Forced vital capacity
IQR: interquartile range
