# Supplementary material for: Age Dependency of GLI Reference Values Compared with Paediatric Lung Function Data in Two German Studies (GINIplus and LUNOKID)
Source: PLoS One. 2016 Jul 20;11(7):e0159678. doi: 10.1371/journal.pone.0159678 (PMC4954644; doi:10.1371/journal.pone.0159678)
Supplement: S1 Fig — (PDF) [file pone.0159678.s001.pdf]

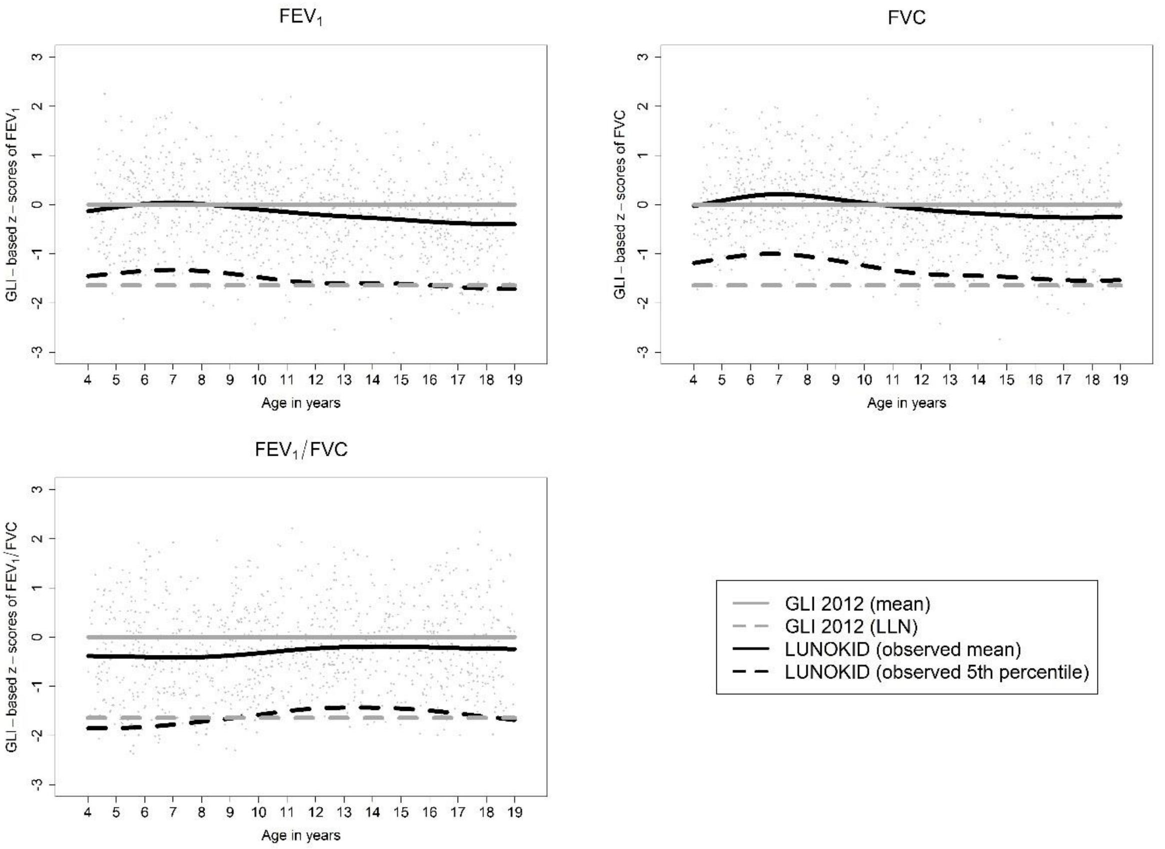

S1 Fig. Comparison of the GLI-z-scores (mean and lower limit of normal (LLN)) with these observed in girls from the LUNOKID data.
