# Supplementary material for: Age Dependency of GLI Reference Values Compared with Paediatric Lung Function Data in Two German Studies (GINIplus and LUNOKID)
Source: PLoS One. 2016 Jul 20;11(7):e0159678. doi: 10.1371/journal.pone.0159678 (PMC4954644; doi:10.1371/journal.pone.0159678)
Supplement: S1 Table — (PDF) [file pone.0159678.s002.pdf]

S1 Table. GINIplus (sensitivity analysis): GLI- and LUNOKID-based z-scores of all lung healthy 15-year old participants (All), participants without a respiratory tract infection in the last 2-4 weeks and participants with no nutritional intervention in the first months of life.

			GLI		LUNOKID	
		**N**	**mean (sd) [a]**	**%≤LLN**	**mean (sd) [a]**	**%≤LLN**
**All (main analysis)**	**FEV_1**	1032	-0.58 (0.87)	11.5% ***	0.02 (1.12) ***	7.3% *
	**FVC**	1029	-0.57 (0.85)	10.9% ***	-0.12 (1.06) ***	8.0% **
	**FEV_1/FVC**	1038	-0.04 (0.97) ***	5.4%	0.28 (1.17) ***	6.2%
**No infection in the last 2-4 weeks**	**FEV_1**	913	-0.59 (0.87)	11.7% ***	0.01 (1.12) ***	7.3%
	**FVC**	911	-0.57 (0.85)	10.9% ***	-0.11 (1.06) ***	7.8% *
	**FEV_1/FVC**	918	-0.04 (0.96) ***	5.1%	0.27 (1.16) ***	6.0%
**No nutritional intervention**	**FEV_1**	549	-0.60 (0.85)	11.7% ***	0.00 (1.09) ***	6.7%
	**FVC**	548	-0.61 (0.85)	12.6% ***	-0.18 (1.07) ***	8.8% *
	**FEV_1/FVC**	551	0.01 (0.97) ***	4.4%	0.33 (1.17) **	5.1%

[a] Two one-sided tests (TOST) for equivalence. Fit is sufficient if the null-hypothesis of a mean z-score outside of the interval [-0.5, 0.5] is rejected (*: p<0.05, **: p<0.01, ***: p<0.001).
